# Natural Autoantibodies in Chronic Pulmonary Diseases

**DOI:** 10.3390/ijms21031138

**Published:** 2020-02-08

**Authors:** Kiyoharu Fukushima, Kazuyuki Tsujino, Shinji Futami, Hiroshi Kida

**Affiliations:** 1 Department of Respiratory Medicine, National Hospital Organization Osaka Toneyama Medical Center, 5-1-1 Toneyama Toyonaka, Osaka 5600045, Japan; fukushima@imed3.med.osaka-u.ac.jp (K.F.); tsujino.kazuyuki.bh@mail.hosp.go.jp (K.T.); 2 Department of Respiratory Medicine and Clinical Immunology, Osaka University Graduate School of Medicine, 2-2 Yamadaoka, Suita, Osaka 5650871, Japan; mm4078fs@gmail.com

**Keywords:** Autoantibody, protein array, idiopathic interstitial pneumonias, sarcoidosis, autoimmune pulmonary alveolar proteinosis, MX1

## Abstract

In autoantibody-mediated autoimmune diseases, pathogenic autoantibodies generated by a failure of central or peripheral tolerance, have different effects mediated by a variety of mechanisms. Interestingly, even non-autoimmune chronic diseases have a set of disease-specific natural autoantibodies that are maintained for a long time. Because most of these natural autoantibodies target intracellular proteins or long non-coding RNAs, they are speculated to be non-pathological and have some important as yet unrecognized physiological functions such as debris clearance. Recently, we revealed a set of disease-specific natural autoantibodies of chronic pulmonary diseases with unknown etiology by protein arrays that enable detection of specific autoantibodies against >8000 targets. Surprisingly, some of the targeted antigens of disease-specific autoantibodies were subsequently reported by other laboratories as strongly associated with the disease, suggesting that these antigens reflect the pathology of each disease. Furthermore, some of these autoantibodies that target extracellular antigens might modify the original course of each disease. Here, we review the disease-specific natural autoantibodies of chronic pulmonary diseases, including chronic fibrosing idiopathic interstitial pneumonias, sarcoidosis, and autoimmune pulmonary alveolar proteinosis, and discuss their utility and effects.

## 1. Introduction

An antibody, also called an immunoglobulin, is a protective protein produced by the immune system in response to the presence of foreign substances, including antigens. The synthesis of antibodies is a vital method in which the adaptive immune system recognizes and responds to external threats. Autoantibodies (autoAbs) are antibodies generated against substances formed by an individual’s body by the failure of central or peripheral tolerance [1]. Generally, autoAbs are not thought to be purposeful products of the human immune system. Many pathological autoantibodies have been recognized as the primary cause of autoimmune diseases by a variety of mechanisms. First, autoAbs can bind to cell surface receptors and cause disease by stimulating or blocking receptor stimulation by natural ligands. For example, autoAbs that bind to the thyroid-stimulating hormone receptor on thyroid cells cause Graves’ disease by stimulating the excessive production of the thyroid hormone by thyroid cells. In myasthenia gravis, autoAbs against the acetylcholine receptor functionally block, alter, or destroy acetylcholine neurotransmission by targeting acetylcholine receptor organization on the postsynaptic neuronal plate. Some autoAbs, such as anti-platelet autoAbs in autoimmune idiopathic thrombocytopenia, induce cell lysis. Other autoAbs function as neutralizing antibodies. For example, autoimmune pulmonary alveolar proteinosis (aPAP), the most common form of PAP (>90% of patients), is caused by the inappropriate production of IgG class autoAbs against granulocyte-macrophage colony-stimulating factor (GM-CSF), a 23-kDa hematopoietic cytokine. Anti-GM-CSF autoAbs are polyclonal and some have high affinity and avidity for their target, thereby completely neutralizing GM-CSF bioactivity on alveolar macrophages preventing them from catabolizing phagocytized surfactants. This results in an accumulation of surfactants in the alveolar airspace and related airspace-occupying consequences [2]. Furthermore, some autoAbs induce inflammation by causing uncontrolled neutrophil activation, such as the anti-neutrophil cytoplasmic antibody (ANCA) in ANCA-associated vasculitis, or causing inflammation at the site of autoantibody binding, such as autoAbs targeting myosin in myocarditis or the anti-cyclic citrullinated peptide antibody in rheumatoid arthritis (RA) [3]. As such, the presence of self-reactive IgG autoAbs in human sera is largely thought to represent a breakdown of central and peripheral tolerance and is typically regarded as a preceding event of autoimmune pathology [3,4]. In addition, the disease environment was reported to promote the cell surface localization of several intracellular antigens in autoimmune diseases such as ANCA-associated vasculitis and RA [5,6].

However, recent studies have shown that sera of patients with or without autoimmune diseases contain IgG autoAbs that react with a set of self-antigens and are unique to each disease [7,8]. These so-called natural autoAbs usually react with intracellular proteins and long non-coding RNAs, and are thought to have evolved as an adaptive mechanism for functions such as debris clearance, which is consistent with their utility as diagnostic and prognostic indicators of disease [8]. In addition, recent research has uncovered the potential role of natural autoAbs in controlling immune responses [9,10,11]. Furthermore, natural autoAbs are sometimes generated against the cell surface or secreted proteins, which might modify the course of the original disease.

**Figure 1:**
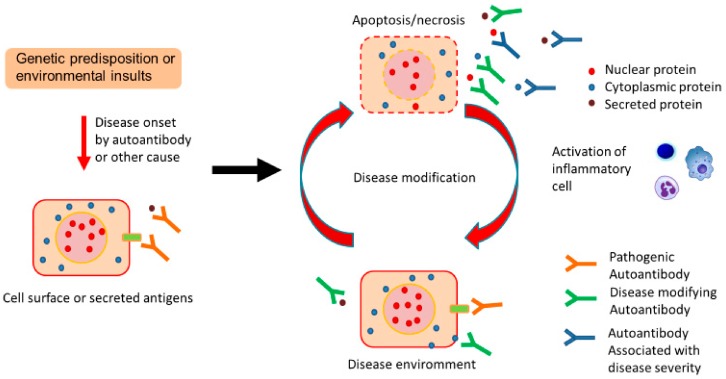
Proposed contribution of natural autoantibodies (autoAbs) to chronic pulmonary diseases. Genetic predisposition or environmental insults trigger disease onset by autoAbs or other causes. Following disease onset, disease-induced tissue damage leads to the increased production of natural autoAbs as an adaptive mechanism to clear debris, and this might promote the cell surface localization of several antigens. Such disease-specific natural autoAbs might reflect ongoing pathology and potentially modify the course of the original disease in association with extracellular or cell surface proteins.

Therefore, although the mechanisms are poorly understood, the disease environment of chronic pulmonary disease might induce increased production of disease-specific natural autoAbs, which would reflect disease status and potentially modify the disease course (Figure 1).

We recently reported the utility of protein array analysis for novel autoAb discovery and deeper insights into disease pathogenesis [12]. Protein arrays consisting of panels of more than 8000 peptides and proteins that include known and candidate autoantigens were originally developed as a powerful tool to study autoimmune disease. In a previous report [12], we performed a comprehensive analysis of serum IgG autoAbs from patients with definitive diagnoses of idiopathic pulmonary fibrosis (IPF), idiopathic non-specific interstitial pneumonia (INSIP), sarcoidosis, and autoimmune pulmonary alveolar proteinosis. This review summarizes the recent insights into natural autoAbs in chronic pulmonary diseases and their potential relevance to the disease course.

## 2. Chronic Fibrosing Idiopathic Interstitial Pneumonias

Interstitial lung disease (ILD) is an umbrella disease consisting of many chronic lung disorders characterized by damage to lung tissues by inflammation and/or fibrosis. In an accurate ILD diagnosis, the differentiation of ILDs such as autoimmune diseases, granulomatous lung diseases, and environment- or drug-induced allergic diseases, leads to the clinical entity of idiopathic interstitial pneumonias (IIPs) [13]. The majority of IIPs are chronic fibrosing IIPs, including idiopathic pulmonary fibrosis (IPF), idiopathic nonspecific interstitial pneumonia (INSIP), and other unclassifiable chronic fibrosing interstitial pneumonias [14]. Circulating autoAbs have been reported to be useful when clustering patients with chronic fibrosing IIPs that lack definitive evidence of causality [15]. For example, chronic fibrosing IIPs with anti-aminoacyl-tRNA synthetase (ARS) autoAbs were reported to show good responses to steroid therapy and have a better prognosis compared with IPF [16]. Another example is the anti-melanoma differentiation-associated gene 5 (MDA5) autoAb that is used to diagnose progressive interstitial lung disease with poor prognosis among chronic fibrosing IIPs [17]. Although some reports support the hypothesis that such autoAbs are pathogenic [18,19], in the absence of direct proof, these autoAbs are thought to represent natural autoantibodies in chronic fibrosing IIPs.

In our previous study, we showed that 17.5% of a cohort of chronic fibrosing IIPs had high titers of serum anti-myxovirus resistance-protein 1 (MX1) autoAbs above the cut-off value, i.e., mean + 4 SD of healthy individuals, by ELISA [12]. MX1 is an important interferon-stimulated gene, which has antiviral activity against a wide range of RNA viruses and some DNA viruses [20]. In the lungs of healthy individuals, MX1 is expressed in Clara cells, alveolar type II pneumocytes, and alveolar macrophages [12]. The expression of MX1 was upregulated in the lungs of patients with chronic fibrosing IIPs and was located in hyperplastic type II pneumocytes and aggregated macrophages in the alveolus. As in the case of anti-ARS autoAbs, it is unlikely that anti-MX1 autoAbs are directly involved in the pathogenesis of chronic fibrosing IIPs because of its intracellular localization. However, anti-MX1 autoAb can be used to cluster a sub-group of patients, whose chronic fibrosing IIPs might originate from different molecular mechanisms, whereby the expression of MX1 is upregulated. Therefore, natural autoAbs, such as anti-MX1 autoAb, might help investigate the pathogenesis of chronic fibrosing IIPs.

Currently, antifibrotic agents are widely used in clinical settings, and the precise classification of chronic fibrosing IIPs is urgently needed to select patients who will benefit from these drugs. To achieve an accurate diagnosis in patients suffering from chronic fibrosing IIPs, histological assessment by surgical or trans-bronchial lung biopsies is required. However, these invasive procedures have the risk of complications and acute aggravation of fibrotic disease. Therefore, such invasive biopsies can be replaced or, at least, supplemented by the assessment of natural autoantibody subsets as a non-invasive lung biopsy, because only 10 μL of serum is needed to detect specific autoantibodies against >8000 targets and differentiate between IPF and INSIP as we previously reported [12]. Natural autoAbs are produced as an adaptive mechanism to clear debris from situation-specific events such as disease pathology, implying that disease-induced tissue damage leads to the increased production of autoAbs appropriate to clear such debris. Therefore, under conditions of ongoing pathology, pathology-specific autoAbs might show selective and measurable titer increases in the blood and can be used as prognostic indicators of disease [11]. Indeed, in chronic fibrosing IIPs, autoAbs were reported to correlate with disease severity and prognosis [21,22].

In our previous study, serum IgGs in IPF and INSIP patients recognized a distinct set of antigens. This finding is promising because if these sets of antigens reflect the molecules that constitute and are upregulated in IPF or INSIP lesions, we can determine which molecular events occur in IPF and INSIP lesions. IPF patient sera reacts with TGF-β-associated molecules (transgelin 2 (TAGLN2), transgelin 3 (TAGLN3) [23], LIM domain-binding protein 2 (LDB2) [24], and HLA complex P5 (HCP5) [25]), regulators of apoptotic pathways (14-3-3 protein zeta/delta (YWHAZ) [26], Trefoil factor 2 protein (TFF2) [27], and RAS-like family 11 member B (RASL11B) [28]), and proteins necessary for cilia formation and maintenance {sperm flagellar 1 (SPEF1) [29], cilia and flagella associated protein 410 (CFAP410)}, and other molecules important for cellular pathways that are also associated with disease development, such as regulating synaptic membrane exocytosis 4 (RIMS4) [30], mitochondrial ribosomal protein S11 (MRPS11) [31], and Ras suppressor-1 (RSU-1) [32]. In INSIP patients, aminoacyl tRNA synthases (glutaminyl-tRNA synthetase (QARS), glycyl-tRNA synthetase (GARS), and methionyl-tRNA synthetase (MARS) [18]), interferon-related molecules (MX1 [12], radical S-adenosyl methionine domain containing 2 (RSAD2) [33], and ninjurin 2 (NINJ2) [34]), and molecules associated with tissue repair (cyclin-dependent kinase 1 (CDK1) [35], retinoid X receptor alpha (RXRA) [36], CDC42 small effector protein 2 (CDC42SE) [37], and poly (ADP-ribose) glycohydrolase (PARG) [38]) are the targets of natural autoantibodies (Table 1).

Interestingly, especially among antigens recognized by INSIP patient sera, there are many extracellular or membrane proteins, which are accessible by antibodies even under normal conditions. This suggests that the antigen-antibody interactions may be implicated in the pathogenesis or clinical course of disease. For example, we found that autoAbs against lysyl oxidase-like 2 (*LOXL2*), an enzyme that promotes crosslinking of extracellular matrix molecules such as collagen and elastin through oxidation, are frequent in INSIP patients. Indeed, a monoclonal antibody against LOXL2 was reported to ameliorate experimental pulmonary fibrosis [42], and humanized IgG4 monoclonal antibody against LOXL2 is under development as a new antifibrotic treatment [43]. We also found an anti-LIM zinc finger domain containing 1 (LMS1) autoAb among INSIP-associated natural autoAbs. Recently, a genomic mismatch in the *LIMS1* gene was identified as a cause of previously unpredictable rejection [44]. In this previous study, production of IgG2- and IgG3-types of anti-LIMS autoAbs was associated with allograft rejection. Voltage-gated hydrogen channel 1 (*HVCN1*) is also interesting, because a deficiency of *HVCN1* was reported to influence antibody production [45] and induce an autoimmune phenotype in mice [46]. In addition to these three proteins, we identified other extracellular or membrane proteins including transmembrane protein 254 (TMEM254), prokineticin 1 (PROK1) [39], and CGRP receptor component (CRCP) [40], as targets of IPF natural autoantibodies. We also identified extracellular or membrane proteins including NINJ2 [41], transmembrane protein 79 (TMEM79) [47], V-Set and transmembrane domain containing 2A (VSTM2A) [48], and fibronectin type III domain containing 4 (FNDC4) [49], as targets of INSIP natural autoantibodies (Table 1).

## 3. Sarcoidosis

Histopathology analysis shows that sarcoidosis is characterized by the presence of non-necrotizing epithelioid and giant cell granulomas. Sarcoidosis is a disease with an incompletely understood pathogenesis that has variable clinical courses [50]. Most frequently, it affects the lungs and lymph nodes. However, multiple organs including eyes, skin, and heart can be affected. For diagnosis, it is necessary to prove the involvement of more than one organ system; but in practice, a combination of the presence of a sarcoid granuloma in one organ and clinical findings of sarcoidosis in another organ is sufficient. Sometimes, diagnosis is difficult because sarcoidosis shares common features with several autoimmune diseases, although typical autoAbs, such as anti-nuclear antibody (ANA) and anti-extractable nuclear antigen antibody (ENA), are usually negative [50]. Therefore, novel biomarkers are needed to distinguish sarcoidosis from other autoimmune diseases.

Because sarcoidosis is a granulomatous disease, we found autoAbs against macrophage-associated antigens, including major facilitator superfamily domain containing 6 (MFSD6) [51] and myocyte enhancer factor 2D (MEF2D) [52] in our analysis of serum autoAbs from patients with a definite diagnosis of sarcoidosis. We also found autoAbs against antigens that were reported to be highly expressed in the granulomatous tissue of sarcoidosis patients, such as von Willebrand factor (vWF) [53] and ferritin heavy chain 1 (FTH1) [54]. Of note, the autoAb against annexin A11 (ANXA11), which is a known susceptibility gene for sarcoidosis, was highly enriched in patients’ sera. *ANXA11*, an important gene for cell division, apoptosis, and neutrophil function [55], is highly expressed in immune cells such as B cells, monocytes, and myeloid cells, and might contribute to the formation of sarcoid granulomas. Furthermore, a non-synonymous single nucleotide polymorphism (SNP) was reported to be associated with sarcoidosis in 490 German patients in a genome-wide association study (GWAS) [56]. In addition, various autoantibodies against membranous and serum proteins, which are easily bound by autoAbs, are frequent in sarcoidosis patient sera. These antibodies (autoAbs against tumor necrosis factor receptor superfamily member 14 (TNFRSF14) [57], growth differentiation factor 10 {GDF10 (BMP3)} [58], mucin-like protein 1 (MUCL1) [59], ring finger and SPRY domain containing 1 (RSPRY1) [60], RRAD and GEM like GTPase 1 (REM1) [61], and gametocyte-specific factor 1-like (GTSF1L) may be modulators of the disease course. These results suggest that autoAbs reflect the ongoing pathophysiology of the disease itself. Therefore, the analysis of autoAbs as biomarkers is useful for diagnosis and prediction of disease progression (Table 2).

Recently, isolated cardiac and central nervous system sarcoidoses, which present primarily as single organ symptoms without clinical evidence of sarcoid involvement in other organs, have been reported [65,66]. Isolated sarcoidosis is difficult to diagnose because the existence of single organ lesions should be detected by single organ manifestations alone. Furthermore, it is difficult to take a biopsy sample from these organs and serum markers such as angiotensin-converting enzyme or soluble interleukin 2 receptor, are not often increased in these patients. However, isolated sarcoidosis was not included in our protein array analysis. Early detection of isolated sarcoidosis is extremely important to improve the prognosis of sarcoidosis patients, and early treatment with corticosteroids and other immunosuppressive agents is desirable. Future studies comparing isolated sarcoidosis are urgently needed because of the difficulty of disease diagnosis and high risk of fatal complications of this disease.

As mentioned above, *ANXA11*, a sarcoidosis-susceptibility gene, is one of the target antigens of natural autoAbs of sarcoidosis. However, *ANXA11* is also an amyotrophic lateral sclerosis (ALS)-associated gene, although the sites of mutation among patients with ALS are different from those found in sarcoidosis patients [62]. Surprisingly, we also identified an autoAb against TAR DNA-binding protein-43 (*TDP-43*), an ALS-associated gene, as a natural autoAb of sarcoidosis. In the motor neurons of ALS patients, the toxicity of cytoplasmic TDP-43 aggregates is suppressed by debranching RNA lariats 1 (DBR1), an RNA lariat debranching enzyme [63]. TDP-43 is an RNA-binding protein, and the mRNA of myocyte enhancer factor 2D (MEF2D) is a target of TDP-43 in ALS patients [64]. Interestingly, both DBR1 and MEF2D are target antigens of natural autoAbs of sarcoidosis. In this regard, comparisons of disease-specific natural autoAbs with those of other diseases may reveal unexpected similarities in the molecular process of the disease.

## 4. Autoimmune Pulmonary Alveolar Proteinosis

Pulmonary alveolar proteinosis (PAP) encompasses chronic lung disorders characterized by the accumulation of surfactant-derived material in pulmonary alveoli, which is accompanied by varying degrees of respiratory insufficiency [67]. Pulmonary surfactants are a complex mixture of phospholipids and proteins secreted by alveolar type II cells and are vital for normal ventilation by preventing collapse of the alveolar structure [68]. They act on the alveolar surface to reduce surface tension and increase lung compliance. Surfactants also contribute to pulmonary innate immunity by opsonizing microbial pathogens, which can lead to the stimulation of phagocytosis and killing of pathogens. A common pathogenic mechanism of PAP is the inability of alveolar macrophages to phagocytize or catabolize surfactants. The most common form of PAP is autoimmune PAP (aPAP, >90% of patients of PAP), which is caused by the inappropriate production of IgG-class autoAbs against GM-CSF, a 23-kDa hematopoietic cytokine [67]. Anti-GM-CSF autoAbs are polyclonal antibodies with different recognition epitopes and functions [69,70,71]. However, not all anti-GM-CSF autoAbs are thought to be involved in the pathogenesis because anti-GM-CSF autoAbs are also detected in healthy individuals [72]. Some forms of these autoAbs have high affinity and avidity to GM-CSF, completely neutralizing the bioactivity of GM-CSF on alveolar macrophages, which blocks alveolar macrophages to catabolize phagocytized surfactants, leading to surfactant accumulation in the alveolus [2,71,73]. In vitro, GM-CSF stimulates the differentiation, proliferation, and survival of granulocytes and monocytes [74,75]. GM-CSF knockout mice develop isolated lung lesions similar to human PAP, which might be caused by the defective clearance of surfactants by alveolar macrophages. Furthermore, spi-1 proto-oncogene (SPI1) is the key down-stream transcription factor of GM-CSF signaling to controlling the differentiation of mouse alveolar macrophages [76] Accordingly, GM-CSF is regarded as a major cytokine in PAP pathophysiology [68]. Because of the reduction or absence of GM-CSF signaling, alveolar macrophages are unable to clear surfactants and also have a less efficient anti-infectious defense system [77]. Furthermore, neutrophil and lymphocyte functions are modified, which explains the tendencies for opportunistic infections in PAP patients [73]. In the context of anti-GM-CSF autoAb production, exogenous GM-CSF administration leads to the production of polyclonal anti-GM-CSF autoAbs with different functions [71,78]. Previous reports have evaluated the activity of each monoclonal GM-CSF autoAb derived from B cells in aPAP patients, and two mechanisms have been considered: neutralization or depletion of GM-CSF, regarding the pathogenicity of anti-GM-CSF autoAbs [70,79]. By clarifying what induces a selective clone of anti-GM-CSF autoAbs to be pathogenic, it may be possible to predict outcomes and discover new drugs.

There are some clinical problems in aPAP. First, anti-GM-CSF autoAbs are polyclonal, and the serum titers of anti-GM-CSF autoAbs in aPAP patients does not correlate with the severity of disease. Thus, the titer of anti-GM-CSF polyclonal autoAbs in blood cannot be used to determine whether aPAP has worsened [80]. Therefore, the severity of the disease is measured by other clinical data such as respiratory symptoms, radiological findings, arterial oxygen partial pressure, pulmonary function test, and six-minute walk test. Patients with aPAP are susceptible to infections and can be affected by various infectious diseases [68,77]. For example, if the lung shadow worsens during the course of aPAP, whether aPAP has deteriorated or other diseases such as infections are involved should be judged “clinically”. Second, the titer of polyclonal anti-GM-CSF autoAbs is not a predictive factor of the clinical course of aPAP patients. Some patients with aPAP improve without treatment, whereas in others, respiratory failure progresses. The factors contributing to the differences between the two groups are still unknown [81]. For these reasons, the titer of anti-GM-CSF polyclonal autoAbs is currently used for diagnosis only. Third, there are only a few treatment options, such as whole lung lavage and inhaled GM-CSF therapy. The high invasiveness of whole lung lavage may be problematic to perform, and inhaled GM-CSF treatment is not standardized because it is currently being investigated in clinical trials and its beneficial effects in terms of clinical outcomes have not been determined [79,82].

GM-CSF is involved in other immune diseases such as RA. GM-CSF is expressed in the synovial membrane and GM-CSF levels are elevated in the synovial fluid of RA patients [83]. Monoclonal antibodies against the GM-CSF receptor or GM-CSF are being evaluated in clinical trials for the treatment of RA inflammation [84]. There have been no reports of cases developing aPAP caused by administration of these antibodies as a therapeutic agent for RA, but one report has shown the appearance of foamy macrophages after administering an anti-GM-CSF receptor alpha monoclonal antibody to cynomolgus monkeys [85]. Interestingly, two cases of aPAP that developed during a course of RA [86] suggest that anti-GM-CSF autoAbs are produced to neutralize the increased GM-CSF and the failure of immune tolerance is essential.

Natural autoAbs against critical molecules in disease pathogenesis and progression are produced frequently in aPAP patients. For example, in our previous study, serum anti-2′-5′-oligoadenylate synthetase 1 (OAS1) autoAbs were abundant in aPAP patients. More recently, mutations of the OAS1 gene in humans were revealed to cause genetic PAP [87]. Furthermore, autoAbs against hematopoietic lineage cell-specific protein (HCLS1), a downstream effector of GM-CSF signaling, were enriched in aPAP patient sera [88]. Furthermore, aPAP patient sera reacted with molecules critical for macrophage functions, such as late endosomal/lysosomal adaptor, MAPK, mTOR activator 1 (LAMTOR1) [89], and plasminogen (PLG) [90]. Other frequently detected autoAbs in aPAP patient sera recognized extracellular proteins important for cellular processes, such as interleukin-10 receptor subunit beta (IL10RB) [91], C-X-C motif chemokine ligand 12 (CXCL12) [92], and bone morphogenetic protein receptor type II (BMPR2) [93]. These autoAbs can readily access antigens and affect the pathophysiology of the disease itself. For example, IL10RB is an essential subunit of the interleukin-10 (IL-10) receptor complex and is indispensable for IL-10 signal transduction [94]. IL-10 is an essential anti-inflammatory cytokine that plays important roles as a negative regulator of immune responses [95,96]. The failure of IL-10 signaling related to mutation in IL-10 receptor was associated with several diseases [97,98,99]. Modulation of IL-10 signaling by autoAbs to IL10RB may alter inflammatory responses in the lung of aPAP patients. CXCL12, has shown to be associated with fibrosis development [100]. Pulmonary fibrosis is present in up to 30% of patients with PAP [101]. Therefore, autoAbs against CXCL12 might be disease modifiers of fibrosis in PAP patients. Finally, bone morphogenetic protein receptor type II (BMPR2) is a serine/threonine receptor kinase that binds to BMPs, which are members of the TGF-β superfamily of ligands involved in paracrine signaling. Balance between BMPs and TGF-β signaling is important for tissue homeostasis [102]. BMPR2 is a central gatekeeper of this balance [103]. AutoAbs against BMPR2 may modulate aPAP disease course by regulating TGF-β responses. Accordingly, the analysis of autoAbs as biomarkers is promising for the diagnosis of disease progression, suggesting antigen-antibody interactions are implicated in the pathogenesis or the clinical course of the disease (Table 3).

For about 50 years since the discovery of natural IgG autoAbs, it was generally thought that they had a low affinity for autoantigens; therefore, the role of natural IgG autoAbs has remained unclear [7]. However, recent studies have revealed that natural IgG autoAbs are not non-reactive, rather they have a fundamental role in systemic innate immune responses [8]. Although direct evidence has not been reported, antigen-antibody interactions of natural autoAbs in the disease context might affect the disease course. Chronic inflammatory conditions including chronic infection or chronic graft versus host disease lead to the development of a variety of systemic autoimmune diseases [104]. Regarding aPAP, toxic effect of occupational exposure on alveolar macrophages might trigger an autoimmune response to produce anti-GM-CSF autoAb [105]. In a large cohort of patients with aPAP, the occurrence of secondary autoimmune diseases was reported [106,107]. Therefore, anti-GM-CSF autoAbs might originate from natural autoantibodies produced under inflammatory conditions by inhalation exposure, and inflammatory conditions caused by anti-GM-CSF autoAb might trigger the secondary autoimmune diseases mediated by the natural autoantibody. Future research on natural autoAbs might elucidate their contribution to regulating ongoing immune processes. The use of state-of-art technologies might enable us to reproduce monoclonal autoAbs from single-B cells, which react with specific autoantigens in a patient’s peripheral blood. Experiments using these monoclonal autoAbs, in vitro and in vivo, will reveal the detailed pathophysiology of natural autoantibodies.

## 5. Conclusions

AutoAbs produced against self-antigens are thought to result from the loss of central or peripheral tolerance triggered by genetic predisposition or environmental insults. AutoAbs are found in a variety of chronic diseases that are not limited to autoimmune diseases. Because some sets of these autoAbs are disease specific, they have clinical potential as diagnostic and prognostic biomarkers. In addition, because some autoAbs recognize extracellular antigens, which are accessible even under healthy conditions, they are considered to potentially modify the pathologies and clinical courses of diseases through a wide range of mechanisms regardless of whether they are causative or symptomatic. Therefore, the identification of these disease-specific autoAbs may provide novel and clinically relevant insights into disease pathologies. Indeed, using protein array analysis we identified molecules that are already known to be critically associated with the pathophysiologies of chronic fibrosing IIPs, sarcoidosis, and aPAP, and which are the targets of natural autoAbs. Currently there is increasing attention on identifying disease-specific autoAbs in various diseases. Protein arrays are a powerful tool, as shown by our data, because they are sensitive, highly reproducible, multiplexed, and provide high throughput data with a minimal sample requirement.

## Figures and Tables

**Table 1:** Subcellular localization of antigens for IPF- and INSIP-specific natural autoAbs enriched by protein array analysis [12].

Intracellular Antigens	Extracellular or Membrane Antigens
IPF TAGLN2, TAGLN3 [23], LDB2 [24], HCP5 [25], YWHAZ [26], TFF2 [27], RASL11B [28], RIMS4 [30], MRPS11 [31], RSU1 [32], PLCG2, CCDC32, SPEF1 [29], CFAP410, ORMDL1, IFI44L, FTSJ1, POLR3K, POLR2L, TCP10L, PHGDH, YTHDF2, METTL21A, METTL14, STK31, NAT6, KCTD14, NIF3L1, CDK9, SEPT4, TIMMDC1, NECAB2, ZNF449, RECQL5, AMOTL2, ROGDI, SUPT4H1	TMEM254, PROK1 [39], CRCP [40]
INSIP QARS, GARS, MARS [18], MX1 [12], RSAD2 [33], NINJ2 [34,41], CDK1 [35], RXRA [36], CDC42SE [37], PARG [38], PEX2, HK1, DCX, ABI1, BUD31, CCDC106, UCMA, ZMAT4, CTSC, TPRXL, NSL1, ALKBH3, ACO2, TCP11L1, NUBPL, ANXA6, TECR, KIF26A, MAPK10, PRKCZ, KCMF1, EIF5, DDI1, RIBC1, PARVA, CYB5R1, TPD52L3, EME1, TBC1D10C, RBFA, SHMT2, GPT2, STK39, MRPL1, PAPSS2	LOXL2 [42,43], LIMS1 [44], NINJ2 [41], HVCN1 [45,46], TMEM79 [47], VSTM2A [48], FNDC4 [49]

**Table 2:** Subcellular localization of antigens for sarcoidosis-specific natural autoAbs enriched by protein array analysis [12].

Intracellular Antigens	Extracellular or Membrane Antigens
Sarcoidosis ANXA11 [55,56,62], TDP-43, DBR1 [63], MEF2D [52,64], Vwf [53], FTH1 [54], RPS6KB2, RIOK3, SGOL1, RAB20, MFSD6 [51], DYNLRB2, TBL1X, BCAS4, CARD14, BCAS4, TCEAL3, MB21D2, C5orf58, PAGE2, PPP2R3B, TIPIN, SRSF8, TCP11, SPATA7, PPA2, PARP16, ANKS3, RFPL1, TCEAL5, SPAG6, REEP1, DAP3, TP53TG1, ME1, HDDC3, RHBDD1, MAD2L1, C18orf8	TNFRSF14 [57], GDF10 BMP3 [58], MUCL1 [59], RSPRY1 [60], REM1 [61], GTSF1L

**Table 3:** Subcellular localization of antigens for aPAP-specific natural autoAbs enriched by protein array analysis [12].

Intracellular Antigens	Extracellular or Membrane Antigens
aPAP OAS1 [87], HCLS1 [88], RHOXF2, SPSB3, KIAA0513, MEIS2, UBE2D2, LINC00663, CDO1, CBX3, HYPK, TSTD2, TPM3, ATRIP, TAB1, BYSL, YPEL1, ZFAND1, AAGAB, NMRK1, INTS3, PIK3R5, NICN1, MGEA5, MRPS7, C9orf78, CNN3, G3BP1, TRIM48, CALB1, IFI16, NFATC2IP, MEIS1, DPCD, PPP1CC, QPRT, PRPF38A, DTYMK, UBE2D2, FLJ25758, PCMTD1, LAMTOR1 [89], BCKDK, RTFDC1, TMOD1, TMEM242, PRUNE2, TPM1, TALDO1	GM-CSF (CSF2) [2,67,68,69,70,71,72,73,74,75,76,77,78,79,80,81,82,83,84,85,86], IL10RB [91], CXCL12 [92,100], BMPR2 [93,102,103], SCAMP3, CYTH3, PLG [90], NPPA
